# Cobalt Neurotoxicity: Transcriptional Effect of Elevated Cobalt Blood Levels in the Rodent Brain

**DOI:** 10.3390/toxics10020059

**Published:** 2022-01-28

**Authors:** Sara Gómez-Arnaiz, Rothwelle J. Tate, Mary Helen Grant

**Affiliations:** 1 Wolfson Centre, Biomedical Engineering Department, University of Strathclyde, Glasgow G4 0NW, UK; sara.gomez-arnaiz@strath.ac.uk; 2 Strathclyde Institute for Pharmacy & Biomedical Sciences, University of Strathclyde, 161 Cathedral Street, Glasgow G4 0RE, UK; r.j.tate@strath.ac.uk

**Keywords:** metal-on-metal (MoM) hip implants, cobalt, systemic cobaltism, neurotoxicity, RNA-Seq, RT-qPCR

## Abstract

Metal-on-metal (MoM) hip implants made of cobalt chromium (CoCr) alloy have shown early failure compared with other bearing materials. A consequence of the abnormal wear produced by these prostheses is elevated levels of cobalt in the blood of patients, which can lead to systemic conditions involving cardiac and neurological symptoms. In order to better understand the implications for patients with these implants, we carried out metal content and RNA-Seq analysis of excised tissue from rats treated intraperitonially for 28 days with low concentrations of cobalt. Cobalt blood levels in dosed rats were found to be similar to those seen in some patients with MoM implants (range: 4–38 μg/L Co in blood). Significant accumulation of cobalt was measured in a range of tissues including kidney, liver, and heart, but also in brain tissue. RNA-Seq analysis of neural tissue revealed that exposure to cobalt induces a transcriptional response in the prefrontal cortex (pref. cortex), cerebellum, and hippocampus. Many of the most up- and downregulated genes appear to correspond to choroid plexus transcripts. These results indicate that the choroid plexus could be the brain tissue most affected by cobalt. More specifically, the differentially expressed genes show a disruption of steroidogenesis and lipid metabolism. Several other transcripts also demonstrate that cobalt induces an immune response. In summary, cobalt exposure induces alterations in the brain transcriptome, more specifically, the choroid plexus, which is in direct contact with neurotoxicants at the blood–cerebrospinal fluid barrier.

## 1. Introduction

Hip arthroplasty procedures successfully lead to the reduction in pain and improved mobility in patients suffering from joint diseases such as osteoarthritis. However, a decade ago, consultants and regulatory bodies reported their concerns over the failures of certain models of metal-on-metal (MoM) hip implants, which resulted in the market withdrawal of the Articular Surface Replacement^TM^ (ASR^TM^) hip implant from DePuy Orthopaedics [1]. Recently, these concerns have been extended to all MoM implant models as it has been demonstrated that MoM implants induce adverse reaction to metal debris (ARMD), which includes metallosis, pseudotumours, aseptic lymphocytic vasculitis associated lesion (ALVAL), and even necrosis [2]. The metal debris is released from the bearing surface, and the taper junction in the case of the total hip replacements (THR), due to wear and corrosion of the metallic parts [3]. Metal ions eventually dissolve and are released into the bloodstream [4]. Co and Cr metal ions in blood are indicative of implant failure and tests for metal ion concentrations should be performed for all patients with MoM implants in the UK according to the MHRA updated guidelines [5]. The European Commission also recommends the monitoring of metal ion levels for MoM implant patients [6].

Neurological conditions thought to be caused by high levels of cobalt ions in blood have been demonstrated in several clinical reports in relation to patients with MoM implants [7,8,9,10,11]. These involve a range of symptoms such as cognitive decline, memory loss, and mood disturbances, in addition to other visual and auditory issues and peripheral neuropathy. There is little information about the actual effects of cobalt in the peripheral and the central nervous system, and the most appropriate response to diminish patients’ symptoms to date is to remove the implant as the source of cobalt ions through revision surgery [8,12,13]. However, the recovery process from cobaltism is not well documented and there is no unified action considered appropriate to treat these patients. Our lack of knowledge on cobalt actions in the body extends further since we also fail to consider the implications for asymptomatic patients with high levels of cobalt in the blood [14].

The effects of cobalt have been extensively researched in vitro with models such as astrocytes [15,16], neurons [17,18,19,20,21], and glial cells [22,23], which are valuable tools to understand the modes of action of cobalt toxicity in the brain. Nevertheless, the nature of in vitro work requires highly controlled experimental conditions, and this can diminish an organism’s complexity. Previous toxicogenomic analyses have demonstrated that in vitro systems fail to fully represent relevant processes occurring in rat liver tissues in vivo after exposure to toxic compounds [24]. Molecular and functional events occurring in vivo at the tissue and organ levels could be crucial mechanisms in the physiological response to cobalt. In this sense, transcriptomic applications have already been proven to be effective in toxicology, not only by understanding the mechanisms, but also by finding early end-points for the detection of toxicity and identification of biomarkers [25]. However, most in vivo cobalt studies have focused on the study of reactive oxygen species and the expression of hypoxia markers. Pregnant female rats dosed orally with 350 mg/L delivered pups with impaired levels of antioxidant proteins in the cerebrum and cerebellum [26]. Caltana et al. observed that the direct cortical injection of cobalt led to histological changes consistent with focal ischaemia involving neuronal and astrocyte morphological changes [27]. Another group applied cobalt dust directly into the dura mater [28] and discovered an elevated expression of proteins involved in thyroid transport and regulation of glycolysis. Nevertheless, it is difficult to establish the relevance of these studies for patients with MoM implants due to the high dosage of cobalt used in the animals, the different types of cobalt delivery methods, and the missing information on the resulting cobalt concentrations in blood or plasma. Our research mimicked the conditions that MoM implant patients endure to obtain a better representation of relevant cobalt toxic mechanisms for them.

The aim of this in vivo work was to identify how cobalt accumulated in tissues after long-term systemic circulation, and specifically answer whether cobalt concentration increased in the brain. For that, we carried out comprehensive time- and dose-response experiments to cobalt treatment. We also sought to understand the underlying molecular changes in the brain at the transcriptional level in laboratory rats after prolonged dose-response exposure. Research on MoM cobalt-induced systemic toxicity is scarce and to our knowledge, there have been few publications addressing neurological manifestations *in vivo*. We hope to generate and test hypotheses through RNA-Sequencing (RNA-Seq) to gain mechanistic insights into the modes of action of cobalt.

## 2. Materials and Methods

### 2.1. Experimental Animals and Research Design

Experiments were performed in adult male Sprague Dawley (SD) rats obtained from Charles River (UK). The body weight range at the start of the experiments was 210–280 g. Food and water were provided *ad libitum*. Their body weight and general aspects of health were monitored daily.

Freshly-made cobalt chloride hexahydrate (CoCl_2_.6H_2_O; Sigma-Aldrich, Dorset, UK) solutions dissolved in distilled water (dH_2_O) and dH_2_O were sterilised through a 0.22 μm syringe-driven filter (Merck Millipore, Watford, UK).

**Figure 1:**
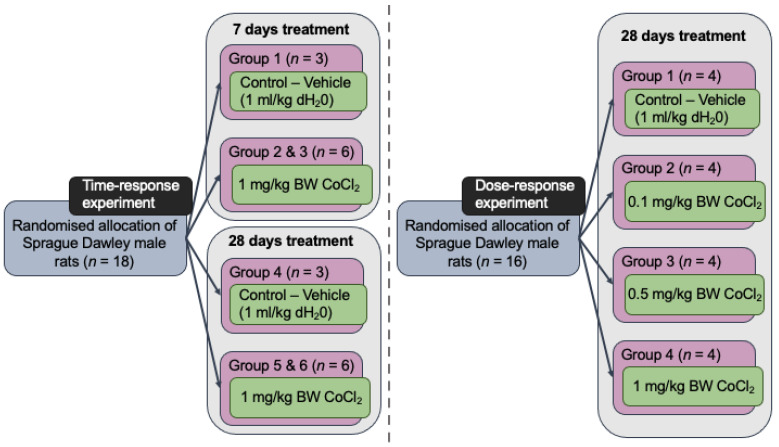
Design of in vivo time- and dose-response experiments showing group distribution and sample size. All injections, both control and Co-treated rats, were carried out intraperitonially.

Two separate in vivo experiments were performed successively. The first experiment was a time-response experiment in which a 7-day cobalt treatment was compared against a 28-day treatment at a fixed cobalt dose. Animals were treated daily with either 1 mL/kg body weight dH_2_O (controls) or 1 mg/kg body weight (BW) CoCl_2_ doses injected i.p. (treated groups). The second in vivo experiment was a dose-response experiment. The animals were given 1 mL/kg dH_2_O i.p. in the case of the control group, or a range of cobalt solutions- 0.1, 0.5, or 1 mg/kg BW i.p. injections for 28 days. Figure 1 depicts the sample size and design information diagrammatically.

### 2.2. Sacrifice of Animals and Tissue Harvest

At the end of the exposure time, animals were killed by carbon dioxide (CO_2_) asphyxiation in a CO_2_ chamber. Each organ or brain part was dissected separately, weighed, and sections of tissue were stored appropriately to preserve its metal content and molecular RNAs. Blood samples were collected through cardiac puncture immediately before death and mixed with 200 µL of heparin (1000 IU/mL diluted 1:10; Sigma-Aldrich, Dorset, UK) to avoid clotting.

### 2.3. Tissue Cobalt Content Measured by ICP-MS

Quantification of cobalt content in all organs was obtained via inductively coupled plasma mass spectrometry (ICP-MS) analysis. For this, 100 mg of tissue from each organ/area of interest was taken and stored at −80 °C until further sample digestion. To obtain a liquid solution of the samples suitable for metal detection, 0.5 mL concentrated nitric acid was used per sample (HNO_3_; TraceSELECT^TM^ Ultra, Sigma-Aldrich (Fluka), Dorset, UK) followed by 0.25 mL 30% hydrogen peroxide (*w*/*w*) (H_2_O_2_; Sigma-Aldrich, Dorset, UK). Each reagent was to left act for 20 min at 100 °C in a hot block to ensure the decomposition of the matrix. A quantity of 0.25 mL was transferred together with 9.75 mL ultrapure water into acid-washed tubes to avoid trace metal contamination. Standard dilutions were prepared from Cobalt Standard for atomic absorption spectrometry stock (TraceCERT^®^, Sigma-Aldrich (Fluka), Dorset, UK). The 1:40 dilution samples were serially introduced to the Agilent 7700× octopole collision ICP-MS system (Agilent Technologies, Cheadle, UK). Scandium or indium was used as the internal standard and data were obtained from the maximum signal with the ICP-MS operating in helium mode.

### 2.4. RNA Extraction

#### 2.4.1. Isolation of RNA

To reduce RNA degradation, 50 or 100 mg from specific segments of brain tissue were dissected and placed in RNase-free tubes with 0.5 mL of RNAlater (Ambion, Life Technologies, Paisley, UK). RNase-free aerosol-resistant filter tips were used for all pipetting steps. Tissues were further cut into thin pieces and completely submerged in the stabilisation solution for overnight storage at 4 °C. RNAlater reagent was removed the next day and the tissues were frozen at −80 °C until RNA extraction.

Tissue was then resuspended in 1 mL QIAzol lysis reagent (Qiagen, Crawley, UK) and a cone ball steel bead was placed inside the tube. The tissue was disrupted with a horizontal Retsch MM200 Mixer Mill (Retsch GmbH, Haan, Germany) set at 30 Hz for 1 min intervals (×3). The lysate was transferred to another tube with 4 mL of QIAzol lysis reagent and the contents vortexed. The pre-processed samples were further prepared according to the protocol of the RNeasy Plus Universal Midi Kit (Qiagen, Crawley, UK).

#### 2.4.2. Quality Check of RNA Samples

RNA purity characterised by the ratio of the absorbances 260/280 nm and 260/230 nm as well as nucleic acid concentration were quantified with a Nanodrop-2000c spectrophotometer (Labtech International, Heathfield, UK). Sample integrity was assessed through microfluidic chip-based analysis (Experion RNA StdSensChip; Bio-Rad, Watford, UK) in the Experion Automated Electrophoresis System (Bio-Rad, Watford, UK) and evaluated with the RNA Quality Indicator (RQI) number [29]. The results of these analyses for pref. cortex, cerebellum, and hippocampus are displayed in Tables S1–S3, respectively, in the Supplementary Materials.

### 2.5. RNA Sequencing (RNA-Seq)

#### 2.5.1. Sample Pooling for RNA-Sequencing

To reduce the RNA-Seq costs, four biological samples for each comparison group were pooled into a single sample. The necessary volume from each sample was adjusted to obtain an RNA quantity of 5–20 μg depending on the brain area yield. The individual sample volumes were pooled into the corresponding RNase-free tubes for each group and the final contents were briefly vortexed.

#### 2.5.2. RNA-Sequencing Analysis by BGI

RNA-sequencing of the pooled samples was performed by BGI Tech Solutions (Hong Kong, China) Co. Ltd. using a BGISEQ-500 sequencing platform with depth of 20 million base pairs (Mb) clean reads per sample and a 50 single-end bases (50SE) read length. Filtering of clean reads and their mapping to the UCSC rn6 rat reference genome were carried out by BGI, in addition to the quantification of gene fold-change.

#### 2.5.3. Gene Ontology (GO) and KEGG Pathway Enrichment Analysis

Further bioinformatic analyses such as hierarchical clustering or generation of Venn diagrams were performed in MATLAB software (The MathWorks Inc. Natick, MA, USA. Release R2021a Update 5, 9.10.0.1739362). The software tool Cytoscape and its plugin ClueGO were used to conduct the Gene Ontology analysis of the DEGs [30,31]. ClueGO allows for a comparison to different reference ontology sets such as Molecular Function (GO MF; 8 April 2016), Biological Process (GO BP; 8 April 2016), and Cellular Component (GO CC; 8 April 2016), which describe specific gene function and cellular location aspects of gene activity as well as the Kyoto Encyclopaedia of Genes and Genomes (KEGG; 14 June 2016) for the specific enrichment of known pathways. Additionally, other online software was used to create protein–protein interaction (PPI) networks of gene-protein products: STRING (https://string-db.org/) [32] (last accessed: 24 December 2020).

### 2.6. Quantitative Real-Time Polymerase Chain Reaction (RT-qPCR)

Real-time quantitative PCR (RT-qPCR) was used to obtain the expression of genes of interest in individual samples in order to validate the RNA-Seq results from the pooled samples. All RT-qPCR experiments were performed in accordance with the minimum information for the publication of quantitative real-time PCR experiments (MIQE) guidelines [33]. The checklist for RT-qPCR assays performed with samples from the in vivo experiments is fully displayed in Table S4 in the Supplementary Materials.

The LunaScript RT SuperMix Kit (New England Biolabs, Hitchin, UK) was utilised to generate cDNA for the dose-response experiment samples. To each RT+ or RT- reaction, enough volume of each sample was added to attain 1 μg RNA in each tube. The thermal cycler (Model 480, Perkin Elmer, Warrington, UK) was pre-heated at 25 °C, and primers were allowed to anneal for 2 min at this temperature, continuing at 55 °C for cDNA synthesis during 10 min, and the final RT inactivation at 95 °C for 1 min. After cooling down the samples, the cDNA samples were stored at −20 °C until further use for RT-qPCR reactions.

The primer sequences were designed to span an intron or exon–exon junction through the NCBI Primer-BLAST tool (https://blast.ncbi.nlm.nih.gov/Blast.cgi) to selectively amplify cDNA (last accessed: 3 March 2020). The primer design criteria followed is displayed in Table S5 in the Supplementary Materials. Oligos were synthesised and purchased from Integrated DNA Technologies (IDT, Leuven, Belgium). The genes with their accession number and primer sequence, amplicon length, and melting temperature are presented in Table S6.

PCR experiments were performed with triplicate RT+ per sample, one no-reverse transcriptase control (RT-) per sample, and one no-template control (NTC) per gene and with the corresponding reference gene controls. The SYBR Green detection method was used for the detection of amplification with the kit PowerUp^TM^ SYBR^TM^ Green Master Mix (Applied Biosystems, Thermo Fisher Scientific, Paisley, UK). The forward and reverse primers (10 pM/μL) and molecular-grade water were mixed with Master Mix (2×). Samples consisting of 1 μL cDNA were pipetted into MicroAmp Fast Optical 96-well reaction plates (Applied Biosystems, Paisley, UK) with the previous mix to a final volume of 20 μL per well. The specific thermal cycling parameters (Table S7) were set according to the optimised PowerUp^TM^ SYBR^TM^ Green Master Mix for fast cycling mode in the StepOnePlus Real-Time PCR system (Applied Biosystems, Paisley, UK). At the end of the cycling process, a melt curve was produced and inspected for the occurrence of primer dimers, misprimes, and possible contamination of genomic DNA.

The method used to calculate the relative fold-change was the comparative C_T_ method [34], with C_T_ being the threshold cycle detected over the 40 run cycles. For gene normalisation, the expression of typical brain reference genes *Ywhaz*, *Tbp*, and *Pes1* was studied. Primer sequences are shown in Table S8. After quantification, the RefFinder web-based tool: https://www.heartcure.com.au/reffinder/ [35] was used to define the most stable reference gene for each tissue (last accessed: 2 March 2020). Full results of RefFinder analyses for each tissue are shown in Figures S1 and S2 in the Supplementary Materials.

### 2.7. Statistics

Shapiro–Wilk and Levene’s tests were used as preliminary methods to evaluate data normality and homogeneity of variances, respectively. Independent samples *t*-test was performed to establish statistical comparisons between the two groups (i.e., control and treatment groups). For statistical comparisons between more groups, the tests selected were one-way analysis of variance (ANOVA) together with the Dunnett’s post-hoc test. Statistical significance was declared when *p*-value < 0.05. The statistical software used was IBM SPSS Statistics 25 (IBM Corp. Armonk, NY, USA. Released 2017. IBM SPSS Statistics for Macintosh, version 25.0).

## 3. Results

### 3.1. Cobalt Accumulates in Organs and in Different Brain Structures

**Figure 2:**
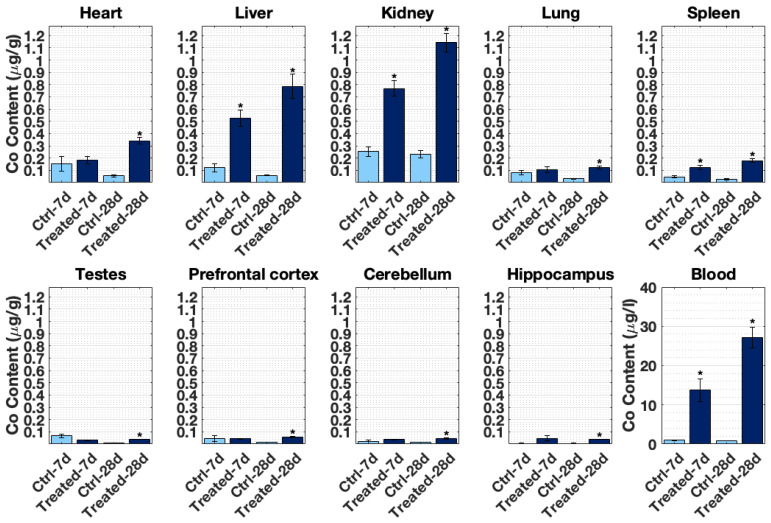
Cobalt content in SD male rats’ tissues (ng/g) and blood (μg/L) at 7- and 28-days of daily i.p. CoCl_2_ injection treatment as assessed by ICP-MS analysis. Control groups were instead injected with distilled water following the same procedures. Figure presents mean ± SEM calculated from *n* = 3 samples in control groups (dH_2_O) and *n* = 6 in treatment groups (1 mg/kg B.W. CoCl_2_). * significantly different between control group and treatment group at a given time-point as assessed by two sample *t*-test (*p* < 0.05).

Figure 2 displays cobalt accumulation in all tissues tested and blood after 7- and 28-day treatments. ICP-MS results reveal that kidney, liver, and heart incorporated the highest Co content among the rats’ organs in that order. After 28 days, pref. cortex, cerebellum, and hippocampus assimilated significant amounts of cobalt (*p* < 0.01, compared with the control rats). This was not the case for the 7-day treatment. Co levels detected in blood from the treated rats by ICP-MS were within the range found in MoM patients. After 28 days, Co detected in rat blood was 27.14 ± 2.70 µg/L compared with the average levels of 1–2 µg/L reported in MoM hip resurfacing patients [36]. The maximum cobalt concentration found in a patient with a THR prosthesis was 6521 µg/L [37].

Several tissues in Figure 2 presented increased cobalt accumulation from 7- to 28-days, indicating possible cobalt time-dependent accumulation in the heart, liver, kidney, spleen, pref. cortex, and blood. However, many of the control samples in the 7-day treatment indicated high levels of cobalt (heart, liver, kidney, lung, spleen, testes, pref. cortex, and cerebellum). This augmentation is likely an artefact of ICP-MS measurements. For values closer to the Co detection limit, it could be that cobalt content is pushed to higher values.

**Figure 3:**
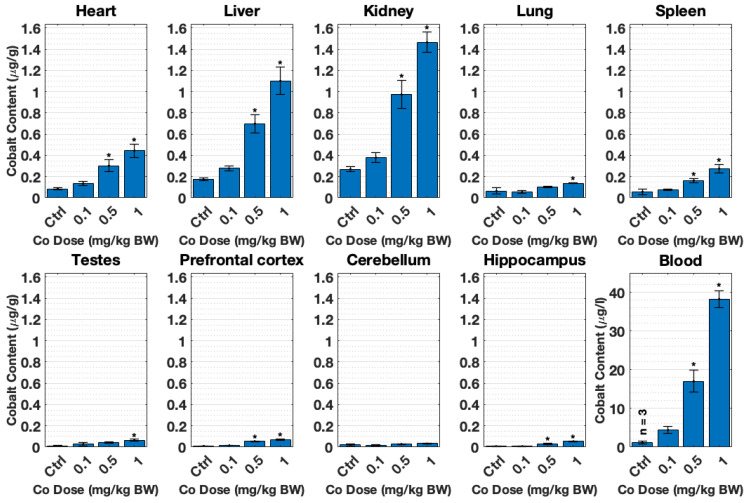
Organ cobalt content (µg/g, tissue; µg/L, blood) obtained by ICP-MS after tissue and blood collection. SD male rats were treated with dH_2_O (control group) or different doses of CoCl_2_: 0.1, 0.5, and 1 mg/kg B.W. Animals were dosed daily with i.p. injections for 28 days. Each group presents mean ± SEM from *n* = 4 rats, and * significant differences in control and treatment means as tested by one-way ANOVA (*p* < 0.05).

Figure 3 shows the cobalt content detected by ICP-MS in all organs after 28 days of cobalt dose-treatment: 0.1, 0.5, and 1 mg/kg B.W. CoCl_2_. This metal content analysis revealed an incremental accumulation of cobalt concentrations in tissues with increased doses. Thus, there is a dose-response accumulation of cobalt, which was significant after 0.5 mg/kg B.W. CoCl_2_ in most tissues. Kidney, liver, and heart in that order accumulated most cobalt in line with the time-response experiment trend (Figure 2). The pref. cortex and hippocampus also accumulated significant levels of cobalt (*p* < 0.01). The same issue was observed with regard to the offset in the control samples, which were closer to the detection limit of the ICP-MS due to their low cobalt content. This effect became obvious in the case of the cerebellum control group.

### 3.2. The Transcriptional Response to the Cobalt Doses Selected Is Non-Proportional

**Figure 4:**
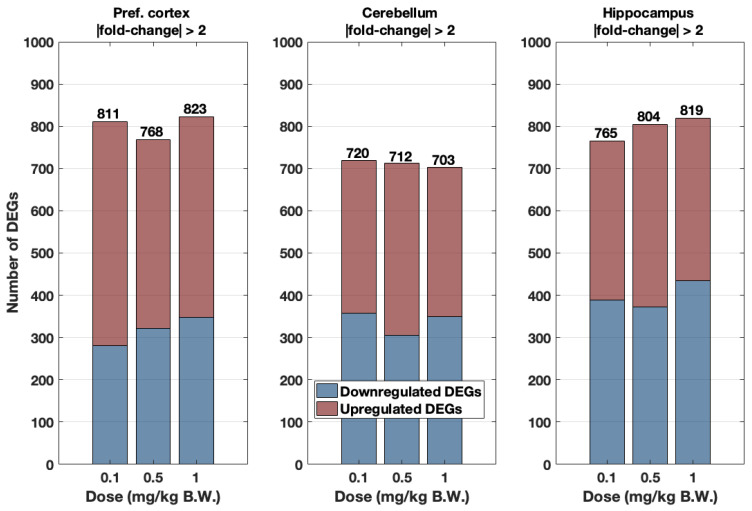
Number of upregulated (red) and downregulated (blue) DEGs (cutoff |fold change| > 2 only) in the pref. cortex, cerebellum, and hippocampus according to cobalt dose treatment: 0.1, 0.5, and 1 mg/kg B.W. CoCl_2_. Animals were dosed i.p. daily for 28 days with those doses or dH_2_O. Data were extracted from RNA-Seq experiments in which *n* = 4 samples were pooled to obtain *n’* = 1, except in the case of the hippocampus treatment group 0.5 mg/kg B.W. CoCl_2_, where *n’* = *n* = 3 as well as for 1 mg/kg B.W. CoCl_2_ where *n’* = *n* = 1.

The cobalt tissue content measured through ICP-MS determined that the pref. cortex and hippocampus of 0.5 and 1 mg/kg BW CoCl_2_-treated groups had significantly greater accumulated cobalt compared to their control groups (Figure 3). However, to evaluate whether the incremental dose cobalt treatment resulted in a progressive transcriptomic response, the number of genes was plotted for all brain areas and the three cobalt treatments: 0.1, 0.5, and 1 mg/kg B.W. CoCl_2_ in Figure 4 (threshold = |fold change| > 2). Although the number of DEGs progressively increased in the hippocampus, there was no clear dose-response in terms of the number of DEGs in the pref. cortex and cerebellum.

**Figure 5:**
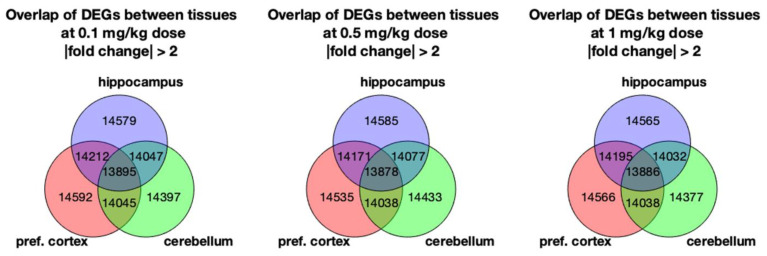
Venn diagrams showing the number of overlapping DEGs between the pref. cortex, cerebellum, and hippocampus at the different cobalt treatment doses: 0.1, 0.5, and 1 mg/kg B.W. CoCl_2_. Rats were treated by daily i.p. injection for 28 days. DEGs were obtained through RNA-Seq by comparing the brain parts’ mRNA abundance of the treatment groups against the controls (dH_2_O-treated).

Figure 5 shows the Venn diagram of DEGs found in the pref. cortex, cerebellum, and hippocampus. The diagram intersections display common DEGs between groups, with the pref. cortex and hippocampus demonstrating a higher number of common genes than the cerebellum for every dose. Although the number of overlapping genes between brain areas indicate that the hippocampus, cerebellum, and pref. cortex are all part of the same tissue (i.e., the brain), the different DEGs also point towards regionalised areas with specific functions. It can also be appreciated that there were few differences in the number of common genes between CoCl_2_ dose regimes. Moreover, it was not possible to identify sets of DEGs that followed a dose-response fold change after hierarchical clustering of the common genes (Figure S3). Thus, we can conclude that the number and range of the doses used does not prompt a dose-response. This does not mean that the transcriptional response elicited is not relevant.

### 3.3. Global Transcriptional Response in the Pref. Cortex and Hippocampus

**Figure 6:**
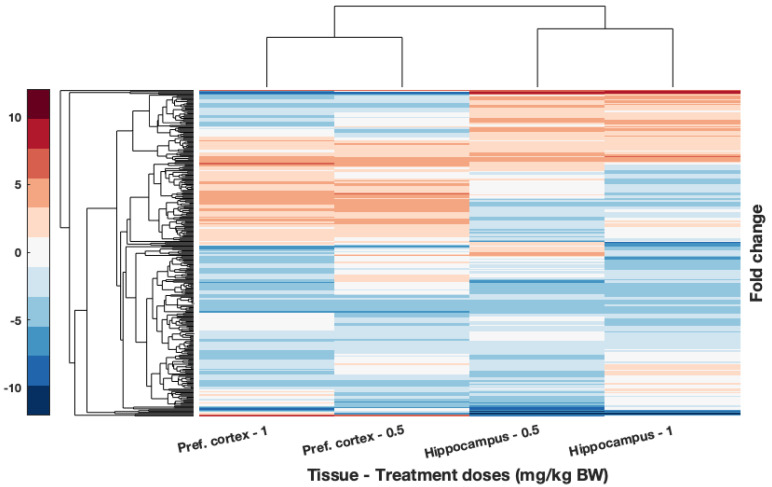
Hierarchical clustering of DEGs from brain tissues with significant accumulation of cobalt: the pref. cortex and hippocampus from rats treated with 0.5 and 1 mg/kg B.W. CoCl_2_. DEGs were obtained from RNA-Seq comparing the RNA isolated from those tissues with those of controls treated with dH_2_O. Condition applied is for fold change to be over 2. Upregulated genes are shown in red while downregulated are displayed in blue. Hierarchical clustering and resulting dendrogram were generated with Euclidian distance. Samples analysed through RNA-Seq were pooled (*n’* = 1) from *n* = 4 pref. cortex samples, *n* = 3 in hippocampus from 0.5 mg/kg B.W. CoCl_2_ treatment group, and *n* = 1 from 1 mg/kg B.W. CoCl_2_ treatment group.

**Figure 7:**
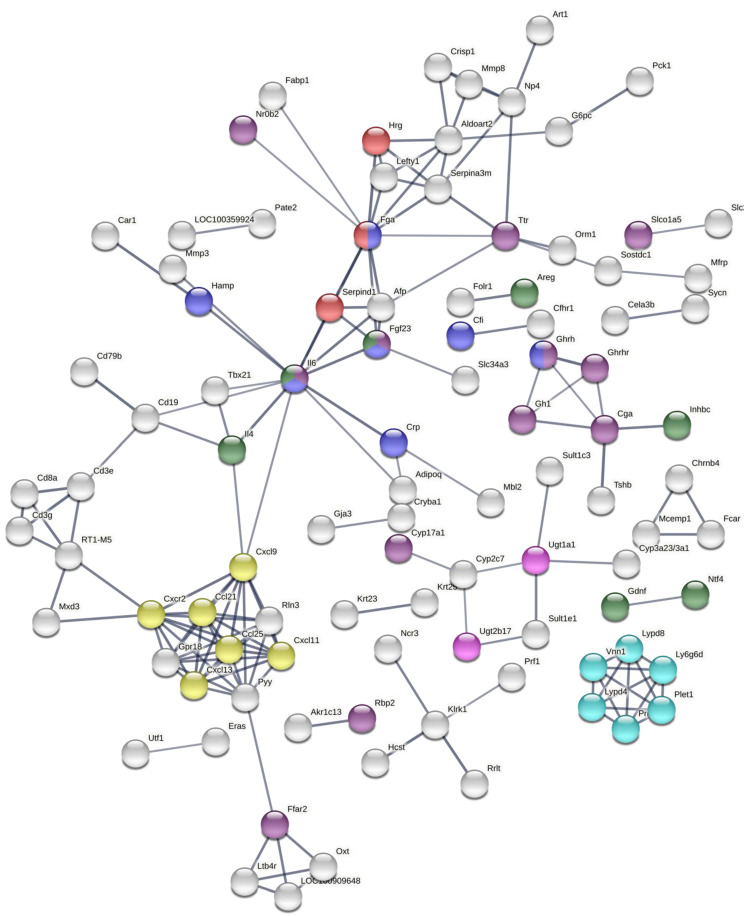
Protein–protein interaction (PPI) network obtained from the STRING web tool by analysing DEGs as their protein products. DEGs were obtained from RNA-Seq analyses of the pref. cortex and hippocampus from rats treated with 0.5 and 1 mg/kg B.W. CoCl_2_ against controls treated with dH_2_O for 28 days of i.p. injections. The following terms/keywords have been highlighted: cellular response to interleukin-6 (blue), regulation of hormone levels (purple), chemokine receptors bind chemokines (yellow), blood coagulation (red), UDP-glucuronosyltransferase activity (pink), growth factor activity (green), and post-translational modification: synthesis of GPI-anchored protein (cyan). The thickness of links between nodes represent the confidence in the interaction, only nodes connected with high confidence (0.7) are displayed.

To further explore the effects on gene expression according to the dosage, this study focused on common DEGs for tissues with significant metal content accumulation. According to the results from the ICP-MS analyses, these tissues are the pref. cortex and hippocampus of rats dosed with 0.5 and 1 mg/kg B.W. CoCl_2_ (Figure 3). The result of this hierarchical clustering is displayed in Figure 6. The gene enrichment analysis of these genes generated with Cytoscape is displayed in Table 1. Several GO terms of importance involved in immunity and hormone activity could be observed. In addition, the protein–protein interaction (PPI) of DEGs–protein products was created to observe the possible links between the overlapped DEGs (Figure 7). The immune axis centred around interleukin-6 (IL6) was clearly separated from other clusters related to growth factors and hormone activity as well as some UDP-glucuronosyltransferases, specifically UGT enzymes that are involved in glucuronidation. Another cluster of interest reflected on the PPI is the glycosylphosphatidylinositol (GPI) anchor biosynthesis.

**Figure 8:**
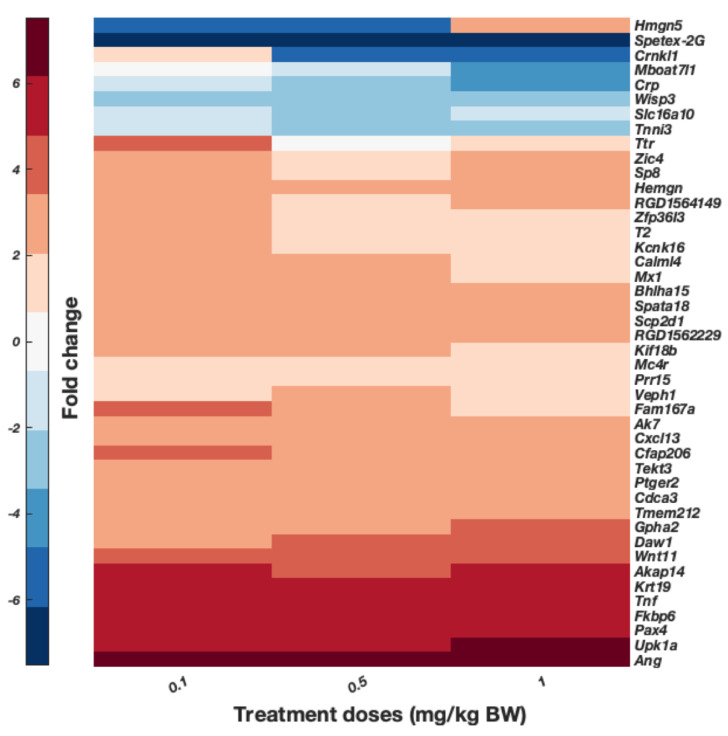
DEGs obtained from the comparison of pref. cortex from rats dosed with 0.1, 0.5, and 1 mg/kg B.W. CoCl_2_ against the control group (dH_2_O). Animals were treated for 28 days with daily i.p. injections. DEGs displayed were obtained from the RNA-Seq analysis of pooled samples (*n’* = 1 from *n* = 4 samples per group). Fold-change gene expression is indicated by colour, as described by the bar in the right side, upregulated genes are displayed in red while downregulated are blue. Genes are displayed as determined by the hierarchical clustering of DEGs over 2-fold-change (*p* < 0.05 from RNA-Seq Poisson distribution), dendrogram not shown (Euclidian distance).

**Figure 9:**
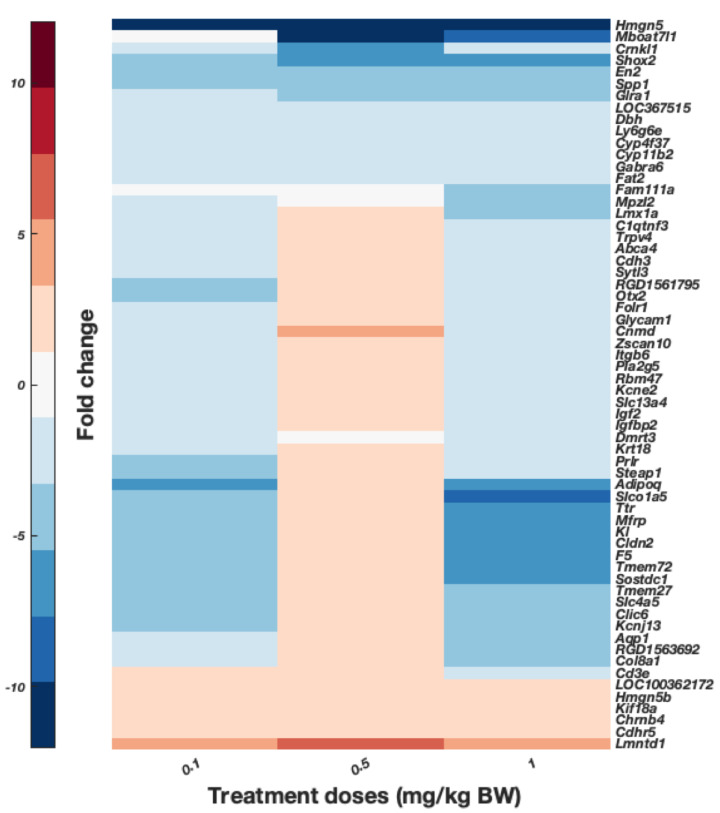
DEGs expressed in hippocampus of rats treated via i.p. with daily injections of 0.1, 0.5, and 1 mg/kg B.W. CoCl_2_ or dH_2_O (control groups) for 28 days. Pooled samples (*n’* = 1 from *n* = 4 samples in group 0.1 mg/kg B.W. CoCl_2_, *n* = 3 from 0.5 mg/kg B.W. CoCl_2_, and *n* = 1 from 1 mg/kg B.W. CoCl_2_ group) were analysed through RNA-Seq and data are presented as the result of hierarchical clustering. DEGs in the graph are only those with fold-change >2 and *p* < 0.05 from RNA-Seq Poisson distribution, dendrogram is not shown. Colour bar presents fold-change: upregulated genes in red and downregulated genes in blue.

To better observe genes possibly involved in cobalt toxic mechanisms, the DEGs expressed over 2-fold change from the pref. cortex and hippocampus were plotted, as shown in Figure 8 and Figure 9, respectively. For display purposes only, DEGs with a significance threshold <0.05 obtained from the Poisson distribution and provided in the original RNA-Seq data are shown. These are displayed as arranged by hierarchical clustering analyses of the DEGs (dendrogram not shown). Figure 8 displays the common genes of the pref. cortex across the three doses, and presents several genes whose proteins have a role in inflammation and immunity such as *Crp*, *Tnf*, and *Cxcl13*. Figure 9 shows significant DEGs of the hippocampus over 2-fold change. Surprisingly, there are several markers attributed to the choroid plexus such as *Clic6*, *Ttr*, *Kl*, *Col8a1*, and others [38,39,40].

A number of genes were evaluated via RT-qPCR according to their high expression level in RNA-Seq data as well as their function. The genes selected were *Tnf*, *Spata18*, *Ttr,* and *Akap14* in the pref. cortex and *Kl* in the hippocampus (see Figures S4 and S5 in the Supplementary Materials). In general, the RT-qPCR gene expression results agreed with the RNA-Seq data, and the fold expression of evaluated DEGs from RNA-Seq and RT-qPCR in this study did correlate. This is consistent with previous high-throughput comparisons between the two technologies [41] as well as research with pooled samples [42]. Thus, it was considered that the RT-qPCR results validate the RNA-Seq results.

## 4. Discussion

### 4.1. Metal Distribution Pattern Is Consistent with Previous Research and Cobalt Accumulates Significantly in the Brain

Figure 2 shows that there is a time-dependent cobalt accumulation trend in most tissues. This time-dependent trend might indicate that the longer cobalt levels remain elevated in blood, with a higher deposition of cobalt in nearly all organs tested, particularly in the heart, liver, and kidney. The accumulation of cobalt in most organs also appears to be proportional to the dose used, as displayed in Figure 3. In addition, cobalt levels were significantly increased in the pref. cortex and hippocampus of rats dosed with 0.5 and 1 mg/kg B.W. CoCl_2_ at 28 days.

The liver, kidney, and heart accumulated more metal than other organs (Figure 2 and Figure 3), which correlates with cobalt organ concentration in humans following cobalt radioisotope distribution [13]. This cobalt distribution follows the same pattern as previous experiments conducted by this research group [43,44] and other teams investigating cobaltism [45]. Most cobalt is excreted through urine [13,46], and given that both kidney and liver are involved in toxin and waste detoxification, it is not surprising that cobalt is predominantly concentrated in these tissues.

With regard to the cobalt in blood after the 28-day treatment with 1 mg/kg B.W., the level was 27.15 ± 2.70 µg/L in the time-response experiment while it was 38.24 ± 2.14 µg/L in the dose-response experiment. Given that the blood cobalt levels were under 100 µg/L, only subtle or moderate neurotoxicity was expected [1].

The cobalt content of two hearts from MoM patients severely affected by systemic cobalt toxicity has been reported in the literature as 4.75 [47] and 8.32 µg/g [48] (reference values for heart cobalt content: 0.06 µg/g) [49]. In addition, other post-mortem analyses on patients with metal-on-polyethylene hip prostheses also revealed significantly elevated cobalt content averaging 0.12 µg/g (range: 0.006–6.299 µg/g) [49]. Table 2 shows a compact presentation of these figures and results for comparison. The higher values of 0.34 ± 0.03 µg/g and 0.44 ± 0.06 µg/g in the rats’ hearts at 28 days with 1 mg/kg B.W. CoCl_2_ treatment were also close to the average content in asymptomatic metal-on-polyethylene (MoP) patients (0.12 µg/g) [49]. Hence, the gradual dosing model used by this study and in previous studies [43] is comparable to the long-term systemic exposure of cobalt through circulating blood in MoM patients.

To our knowledge, there is no information on the cobalt concentrations of MoM patients in the brain, probably due to the difficulty of obtaining samples from patients. In this study, the pref. cortex, and hippocampus had significant cobalt accumulated at 28 days (Figure 2 and Figure 3). A study by Apostoli et al. with rabbits dosed with cobalt for 18 days intravenously reported elevated brain levels, 0.2 ± 0.2 µg/g, from the average control levels, 0.06 ± 0.04 µg/g dry weight, and cobalt whole blood, 420.9 ± 154.5 µg/L (see Table 2 for reference [45]). The histology in several organs only reported damage to the eyes and the auditory systems. The model used for this study had much lower cobalt blood concentration and animals remained in good health, while Apostoli et al. described balance disturbance in rabbits due to vestibular damage. Given the literature and observations of this study, cobalt appeared to induce only subtle or moderate neurotoxicity in this study even when cobalt had significantly accumulated in the rats’ brains.

### 4.2. The Low Range Cobalt Dosage Used Does Not Lead to a Dose-Response

No dose-response was demonstrated either in the number or in the average fold change of DEGs elicited by cobalt treatment (Figure 4 and Figure S3). Previous studies suggest that the fraction of ionic cobalt remains constant throughout a wide range of cobalt concentrations in the blood due to its albumin binding capacity [13]. If cobalt is being sequestered by albumin, it is likely that the response to any administered cobalt treatment will be dampened. However, it is also possible for cobalt toxic effects to follow a dose response, but the few concentrations used here did not cover such a range of toxicity.

### 4.3. Overall Transcriptional Effects of Cobalt and the Choroid Plexus as a Target of Cobalt Toxicity

This study found some GO terms of interest that could be associated with cobalt toxicity in Table 1. This is the case of ‘steroid hormone biosynthesis’, which is comprised of a few genes of the Cytochrome P450 (*Cyp* prefix) family as well as by a couple UDP-glucuronosyltransferases (*Ugt* prefix) and a sulfotransferase (*Sult* prefix). The protein products of these gene families form part of the drug metabolism and detoxification pathways. CYPs are also involved in the biosynthesis of serotonin, dopamine [51], and steroid hormones [52]. The findings suggest that CYPs could be a target of cobalt since they normally bind to the heme metal substrate as some metal ions have been observed to inactivate members of the CYP family [53]. Nevertheless, there are other large GO terms linked with hormone homeostasis such as ‘regulation of hormone levels’, ‘steroid binding’, and ‘hormone activity’ in Table 1. Some of these are not directly related to steroid hormones e.g., essential thyroid related genes (*Tshb*), whose deletion leads to hypothyroidism, and other GO terms such as ‘response to retinoic acid’. Thus, nuclear receptors could possibly be regulated by cobalt. Nuclear receptors may bind steroids, retinoic acid, or thyroid hormones, and this binding depends on the nuclear receptor zinc finger domain. The CYP family synthesises some of the ligands that the nuclear receptors bind to, and further experiments could be performed to ascertain whether cobalt binds to CYPs or to nuclear receptors [54].

The PPI analysis (Figure 7) displayed a regulation of hormone levels consistently with the GO enrichment analysis as well as a network of drug-metabolising enzymes consisting of a few *Cyp*, *Ugt*, and *Sult* genes. The immune and haematopoietic axis centred around *Il6* with important chemokine presence can also be observed. This immune response was also reflected in the GO enrichment from Table 1. Some of the immune-related GO terms are ‘response to interleukin-6′, ’T-helper 17 cell lineage commitment’, ‘response to interleukin-1′, ‘cell chemotaxis’, and ‘cytokine receptor binding’, which suggest immune cell differentiation, activity, and migration. Other factors related to blood coagulation such as *Hrg*, *Serpind1*, and *Fga* are displayed in Table 1. Activation of the immune system and dysregulation of haematopoietic transcriptional programmes could lead to several autoimmune and blood disease syndromes. Cobalt was a historical treatment of anaemia, and polycythaemia and skin rashes have been documented as a sporadic result of cobalt treatment [13]. Finally, the PPI also reported the presence of ‘GPI-anchored protein’ related transcripts. This is a post-transcriptional modification that mainly occurs in the endoplasmic reticulum, where most glycosylphosphatidylinositol (GPI) synthesis proteins function [55]. GPI-anchored protein synthesis depends on phospholipids, while the synthesis of steroid hormones is determined by cholesterol, hence, it is suggested here that cobalt modulates lipid metabolism. It is possible that cobalt could modulate lipid metabolism directly as cobalt has been seen to affect the rigidity of lipid membranes such as liposomes [56]. Lipid droplets were present in the spleen of a patient with a CoCr prosthesis [57]. Moreover, intracytoplasmic lipid and lipofuscin accumulation were found in a recent heart biopsy of a patient with arthroprosthetic cobaltism [48].

This study also found genetic markers in the pref. cortex (Figure 8) and hippocampus (Figure 9) almost exclusively attributable to the choroid plexus (e.g., *Clic6*, *Klotho* (*Kl*), transthyretin (*Ttr)*, *Veph1*, some cilia markers, and *Scl* transporters). The molecular characterisation of the choroid plexus has only been achieved recently [38], and unfortunately the GO ontologies have not been adequately updated to report its presence. The choroid plexus is anatomically attached to the hippocampus and its joint dissection can go unnoticed when doing a fast isolation, as reported by specialists in the choroid plexus [39,58]. In fact, several studies investigating the effect of drugs or other interventions in the hippocampus have knowingly or unwittingly reported choroid plexus markers [58,59,60]. There are also markers of the choroid plexus such as transthyretin (*Ttr*) in the pref. cortex, and it is possible that part of the choroid plexus has also been included in brain samples other than the hippocampus, since the choroid plexus is distributed through all brain ventricles [58]. Different studies have revealed that heavy metals preferentially accumulate early on in the choroid plexus, which appears to retain them, thus protecting the brain [61,62,63,64,65,66]. Harrison-Brown et al. discovered that the penetration of cobalt in the cerebrospinal fluid (CSF) of MoM patients was limited to 15% of the cobalt in plasma [67]. They also found a nonlinear trend with a ceiling effect in the CSF cobalt accumulation in relation to Co plasma levels in blood. Thus, the choroid plexus could function as an absorptive barrier, and early cobalt accumulation and damage in the brain might occur in the choroid plexus.

The choroid plexus is also a place for steroid hormone biosynthesis [68] and it hosts metabolising enzymes to deal with and metabolise xenobiotics [69]. Many immune cells are resident in the choroid plexus, which works as the site for immune trafficking with the brain [70]. B and T lymphocytes can infiltrate the choroid plexus and affect its function under certain challenges, thus leading to inflammation [71,72,73]. In particular, the ‘cell chemotaxis’ GO term includes *Cxcl13*, a chemokine that recruits B lymphocytes, and it has been involved in lymphoid infiltration in the choroid plexus in a mouse model of neuropsychiatric lupus [73]. Moreover, very recently, modulation of *Otx2* expression in the choroid plexus has been seen to regulate anxiogenic behaviour in mice [74], and in two studies, the choroid plexus transcriptome quickly responded to stress tests in mice [39,58]. Given that the choroid plexus has been implicated in depression disorders [75] and that some patients with elevated cobalt levels in their blood showed signs of neuropsychiatric symptoms such as depression [8,10], one might speculate that cobalt toxicity in the choroid plexus could impair its function, and contribute towards mood dysregulation.

## 5. Conclusions

In summary, although the rat pref. cortex and hippocampus accumulated lower amounts of cobalt than other tissues, these accumulations were still significant at similar elevated circulating cobalt levels to those found in some patients with MoM implants. We found that the common transcriptional response to cobalt in the brain areas analysed involved hormone and drug-metabolising activity, in addition to also describing a powerful immune response, perhaps mediated by inteleukin-6 (IL-6). An underlying dysfunction in lipid metabolism is also likely. We suggest that these mechanisms could be instigated as a consequence of cobalt ion binding and substitution of native metal ions of CYPs or nuclear receptors. In the future, researchers should consider evaluating the markers of inflammation and lymphoid cell activation as well as the steroidogenic activity in the choroid plexus in response to cobalt. Thus, we have generated a mechanistic hypothesis for cobalt neurotoxicity that could be explored further and have relevant implications for patients with MoM implants who develop neurological health issues.

## Figures and Tables

**Table 1:** Enriched GO terms obtained from DEGs of the pref. cortex and hippocampus in response to cobalt treatment with 0.5 and 1 mg/kg B.W. CoCl_2_ compared to control animals (dH_2_O). Rats were dosed for 28 days with i.p. injections. GO terms annotated were significantly enriched with *p* < 0.05. GO terms enriched belong to the Molecular Function (MF; 8 April 2016), Biological Process (BP; 8 April 2016), Cellular Component (CC; 8 April 2016), and KEGG (14 June 2016) GO databases.

Gene Ontologies (GO) and GO Terms	Number of Genes with Annotations in the Ontology (%)	Number of Genes Represented in GO Terms (%)
**Biological Process (BP) GO terms** (8 April 2016)	194 (72.12%)	93 (34.57%)
Regulation of hormone levels	*Afp, Bco1, Bik, Cga, Cyp11a1, Cyp17a1, Fam3b, Ffar2, Fga, Fgf23, Gh1, Ghrh, Ghrhr, Il6, Nr0b2, Nt5c1b, Pax8, Slc30a8, Slco1a5, Sult1e1*	
Response to interleukin-6	*Crp, Fga, Fgf23, Ghrh, Hamp, Il6, Pck1*	
T-helper 17 cell lineage commitment	*Batf, Il6, Ly9*	
Response to vitamin	*Cyp11a1, Fgf23, Folr1, Hamp, Orm1, Otc, Sult2a1, Tshb*	
Response to pH	*Acer1, Gh1, Gja3, Kcnk18, Pck1*	
Response to interleukin-1	*Ccl21, Ccl25, Cyp11a1, Il6, Mmp3, Pck1, Slc30a8*	
Cell chemotaxis	*Ccl21, Ccl25, Ccr6, Cxcl13, Cxcl9, Cxcr2, Ffar2, Hrg, Stap1*	
Organ formation	*Folr1, Foxh1, Gdnf, Ntf4, Pax8*	
Cell fate commitment	*Batf, Elf5, Gata5, Gsx1, Gsx2, Il6, Ly9, Myl2, Ntf4, Olig3, Sostdc1*	
**Molecular Function (MF) GO terms** (8 April 2016)	185 (68.77%)	60 (22.3%)
Cytokine receptor binding	*Bmp10, Ccl21, Ccl25, Cxcl13, Cxcl9, Gh1, Il6, Inhbc, Ntf4, Stap1*	
Heparin binding	*Ang2, Comp, Cxcl13, Hrg, Mcpt4, Serpind1, Wisp3*	
Growth factor activity	*Areg, Bmp10, Fgf23, Gdnf, Il6, Inhbc, Ntf4*	
Steroid binding	*Comp, Crp, Cyp11a1, Fabp1, Sult1e1, Ugt1a1*	
Hormone activity	*Bmp10, Cga, Gh1, Ghrh, Gpha2, Hamp, Inhbc, Pyy, Rln3, Tshb*	
**KEGG terms** (14 June 2016)	97 (36.06%)	29 (10.78%)
Steroid hormone biosynthesis	*Cyp11a1, Cyp17a1, Cyp2c7, Cyp3a23/3a1, Sult1e1, Ugt1a1, Ugt2b17*	
Hematopoietic cell lineage	*Cd19, Cd3g, Cd8a, Fcer2, Il1r2, Il6*	
**Cellular Component terms** (8 April 2016)	216 (80.3%)	18 (6.69%)
External side of plasma membrane	*Cd19, Cd8a, Cxcl9, Fcer2, Fga, Folr1, Hyal5, Il6, Itgad, Trpm8*	

**Table 2:** Cobalt concentrations of whole blood (WB), brain and heart tissues in the dose and time-response experiments and other studies that mimic gradual cobalt release through daily treatment [45], cobalt tissue analyses in the cardiac tissue, and serum of MoM patients with cobaltism [47,48] as well as post-mortem heart tissue from metal-on-polyethylene (MoP) patients [49]. Cobalt content values in unexposed human brain (<0.025 µg/g), heart (0.060 µg/g) and blood (<1 µg/L) were obtained from [36,49,50] in that order. The abbreviations are: i.p., intraperitoneal injections; WB, whole blood; avg., average; C, control; T, treatment; THA, total hip arthroplasty. * significantly different control and treatment groups as assessed by one-way ANOVA with Dunnett’s multiple comparison.

Studies		Time-Response Study	Dose-Response Study	[45]	[47]	[48]	[49]
Study outline		CoCl_2_	CoCl_2_	CoCl_2_			MoP Patients
		SD rats	SD rats	rabbits	MoM patient	MoM patient	
		28 days	28 days	18 days			
		1 mg/kg BW	1 mg/kg BW	1354 µg/mL			
		i.p.	i.p.	Intravenous infusion			
Tissues		*n* = 3 (C) and 6 (T)	*n* = 4 (C) and 4 (T)	*n* = 2 (C) and 4 (T)	case report	case report	*n* = 73 (C), 75 (THA)
WB or serum (µg/L)	Cobalt exposed	27.15 ± 2.70 * (WB)	38.24 ± 2.14 * (WB)	420.9 ± 154.5 (WB)	192 (serum)	287.6 (serum)	Unknown
	Controls/unexposed	0.87 ± 0.00	1.17 ± 0.32	11.7 ± 2.7	<1		
Brain (µg/g)	Cobalt exposed	0.06 ± 0.00 * (pref. cortex)	0.07 ± 0.01 * (pref. cortex)	0.2 ± 0.2	Unknown		
	Controls	0.02 ± 0.00	0.01 ± 0.00	0.06 ± 0.04	<0.025		
Heart (µg/g)	Cobalt exposed	0.34 ± 0.03 *	0.44 ± 0.06 *	0.7 ± 0.5	4.75	8.32	0.12 (avg.); range: 0.006–6.299
	Controls/unexposed	0.06 ± 0.01	0.09 ± 0.01	0.07 ± 0.1	0.06		

## Data Availability

All data underpinning this publication are openly available from the University of Strathclyde KnowledgeBase at https://doi.org/10.15129/cbbe122f-4ee1-4bfb-9ec7-46c664bf10f2 (accessed on 29 November 2021).

## Supplementary Materials

The following are available online at https://www.mdpi.com/article/10.3390/toxics10020059/s1, Table S1. Quality check of RNA samples from the pref. cortex tissue obtained from the dose-response in vivo experiments, Table S2. Quality check of RNA samples from the cerebellum tissues obtained from the in vivo dose-response experiments, Table S3. Quality check of RNA samples from the hippocampus tissues obtained from the in vivo dose-response experiments, Table S4. MIQE checklist from the MIQE guidelines for reproducibility and assessment of experimental RT-qPCR conditions, Table S5. Criteria for the design of the primers selected through NCBI Primer-BLAST tool, Table S6. Primer sequences of targeted genes designed for the in vivo dose-response experiment, Table S7. Fast PCR thermal cycling steps based on PowerUp^TM^ SYBR^TM^ Green Master Mix instructions for StepOnePlus Real-Time PCR system, Table S8: Primer sequences of control genes, Figure S1. RefFinder ranking of three reference genes Ct values in pref. cortex samples from the in vivo dose-response experiment, Figure S2. RefFinder ranking of three reference genes Ct values in hippocampus samples from the in vivo dose-response experiment, Figure S3. Hierarchical clustering of DEGs over 2-fold-change from RNA-Seq data obtained from the comparison of the pref. cortex, cerebellum, and hippocampus of rats treated with three concentrations of cobalt against those of controls treated with dH_2_O, Figure S4: Fold change mRNA gene expression levels obtained from RNA-Seq and RT-qPCR, Figure S5: Quantification of *Tnf*, *Spata18*, *Ttr*, and *Akap14* delta (CT) values (CT target gene–CT internal control) in the pref. cortex and *Kl* in the hippocampus through RT-qPCR.
