# Supplementary material for: Cobalt Neurotoxicity: Transcriptional Effect of Elevated Cobalt Blood Levels in the Rodent Brain
Source: Toxics. 2022 Jan 28;10(2):59. doi: 10.3390/toxics10020059 (PMC8878729; doi:10.3390/toxics10020059)
Supplement: Supplementary file 1 [file toxics-10-00059-s001.zip › toxics-1511917-supplementary.pdf]

toxics

Supplementary Materials: Cobalt Neurotoxicity: Transcriptional Effect of Elevated Cobalt Blood Levels in the Rodent Brain

Sara Gómez-Arnaiz, Rothwelle J. Tate and Mary Helen Grant

Table S1. Quality check of RNA samples from the pref. cortex tissue obtained from the dose-response *in vivo* experiments. Parameters shown are RNA concentration (ng/µl), and ratios [260/280] and [260/230] indicating RNA purity, in addition to ratio [28S/18S] and RQI indicating RNA integrity. The bottom part of the table shows the pooled samples. 'Crtx X.Y' abbreviation refers to pref. cortex sample number Y from group X (group 1 is the control group which had daily dH_2O i.p. injection treatment; 2, 3 and 4 treatment groups had 0.1, 0.5 and 1 mg/kg B.W $CoCl_2$ daily i.p. injections for 28 days respectively). There are n = 4 samples per each control or treatment group, each group was later pooled into a single sample for RNA-Seq evaluation.

Quality Check of RNA Samples from the Pref. Cortex					
**Samples**	**RNA Concentration (ng/µL)**	**Ratio [260/280]**	**Ratio [260/230]**	**Ratio [28S/18S]**	**RQI**
**Pref. cortex Control (Crtx 1.1)**	237.3	2.08	2.16	1.30	9.4
**Pref. cortex Control (Crtx 1.2)**	309.3	2.08	2.30	1.59	9.6
**Pref. cortex Control (Crtx 1.3)**	208.9	2.06	2.27	1.39	9.7
**Pref. cortex Control (Crtx 1.4)**	278.0	2.07	2.25	1.34	9.7
**Pref. cortex 0.1mg/kg BW (Crtx 2.1)**	410.8	2.08	2.24	1.30	9.3
**Pref. cortex 0.1 mg/kg BW (Crtx 2.2)**	178.0	2.07	2.02	1.53	9.2
**Pref. cortex 0.1 mg/kg BW (Crtx 2.3)**	375.2	2.09	2.21	1.53	9.4
**Pref. cortex 0.1 mg/kg BW (Crtx 2.4)**	437.9	2.09	2.26	1.17	8.9
**Pref. cortex 0.5 mg/kg BW (Crtx 3.1)**	260.2	2.06	2.29	1.31	9.3
**Pref. cortex 0.5 mg/kg BW (Crtx 3.2)**	256.6	2.09	2.20	1.35	9.2
**Pref. cortex 0.5 mg/kg BW (Crtx 3.3)**	291.0	2.07	2.24	1.42	9.5
**Pref. cortex 0.5 mg/kg BW (Crtx 3.4)**	293.0	2.09	2.25	1.54	9.5
**Pref. cortex 1 mg/kg BW (Crtx 4.1)**	282.7	2.07	2.27	1.34	9.7
**Pref. cortex 1 mg/kg BW (Crtx 4.2)**	279.2	2.09	1.98	1.43	9.3
**Pref. cortex 1mg/kg BW (Crtx 4.3)**	308.9	2.11	1.32	1.47	9.3
**Pref. cortex 1 mg/kg BW (Crtx 4.4)**	352.4	2.06	2.18	1.58	9.7
**Pooled Samples**					
**Pref. cortex - Control**	252.1	2.06	2.22	1.38	9.5
**Pref. cortex - 0.1 mg/kg BW**	280.7	2.06	2.15	1.41	9.0

**Pref. cortex - 0.5 mg/kg BW**	272.2	2.06	2.22	1.25	8.5
**Pref. cortex - 1 mg/kg BW**	260.4	2.06	1.90	1.34	9.5

Table S2. Quality check of RNA samples from the cerebellum tissues obtained from the *in vivo* dose-response experiments. Parameters shown are RNA concentration (ng/µl), and ratios [260/280] and [260/230] indicating RNA purity, in addition to ratio [28S/18S] and RQI indicating RNA integrity. The bottom part of the table shows the pooled samples. 'Crbm X.Y' abbreviation refers to cerebellum sample number Y from group X (group 1 is the control group with daily i.p injections of dH_2O; and 2, 3 and 4 treatment groups had 0.1, 0.5 and 1 mg/kg B.W $CoCl_2$ daily i.p. injections for 28 days respectively). There are n = 4 samples per each control or treatment group, each group was later pooled into a single sample for RNA-Seq evaluation.

Quality Check of RNA Samples from the Cerebellum					
**Samples**	**RNA concentration (ng/µl)**	**Ratio [260/280]**	**Ratio [260/230]**	**Ratio [28S/18S]**	**RQI**
**Cerebellum Control (Crbm 1.1)**	199.8	2.06	2.24	1.44	9.7
**Cerebellum Control (Crbm 1.2)**	360.8	2.10	2.31	1.64	9.9
**Cerebellum Control (Crbm 1.3)**	307.8	2.08	2.13	1.45	9.6
**Cerebellum Control (Crbm 1.4)**	303.9	2.08	2.30	1.45	9.7
**Cerebellum 0.1 mg/kg BW (Crbm 2.1)**	365.6	2.03	2.21	1.46	9.7
**Cerebellum 0.1 mg/kg BW (Crbm 2.2)**	366.4	2.07	2.25	1.59	9.9
**Cerebellum 0.1 mg/kg BW (Crbm 2.3)**	351.2	2.09	2.28	1.49	9.3
**Cerebellum 0.1 mg/kg BW (Crbm 2.4)**	283.6	2.06	2.22	1.48	9.8
**Cerebellum 0.5 mg/kg BW (Crbm 3.1)**	330.2	2.09	2.32	1.55	9.6
**Cerebellum 0.5 mg/kg BW (Crbm 3.2)**	354.6	2.09	2.29	1.66	9.8
**Cerebellum 0.5 mg/kg BW (Crbm 3.3)**	388.9	2.09	2.24	1.57	9.8
**Cerebellum 0.5 mg/kg BW (Crbm 3.4)**	221.3	2.06	2.28	1.74	8.3
**Cerebellum 1 mg/kg BW (Crbm 4.1)**	454.5	2.09	2.25	1.38	9.1
**Cerebellum 1 mg/kg BW (Crbm 4.2)**	372.7	2.09	2.31	1.41	9.7
**Cerebellum 1mg/kg BW (Crbm 4.3)**	345.3	2.10	1.88	1.58	9.8
**Cerebellum 1 mg/kg BW (Crbm 4.4)**	534.3	2.09	2.31	1.49	9.6
**Pooled Samples**					
**Cerebellum - Control**	278.7	2.07	2.22	1.35	-
**Cerebellum - 0.1 mg/kg BW**	309.5	2.08	2.25	1.43	9.4
**Cerebellum - 0.5 mg/kg BW**	300.8	2.08	2.25	1.53	9.8
**Cerebellum - 1 mg/kg BW**	401.1	2.08	2.14	1.52	9.6

Table S3. Quality check of RNA samples from the hippocampus tissues obtained from the *in vivo* dose-response experiments. Parameters shown are RNA concentration (ng/µl), and ratios [260/280] and [260/230] indicating RNA purity, in addition to ratio [28S/18S] and RQI indicating RNA integrity. The bottom part of the table shows the pooled samples. 'Hppc X.Y' abbreviation refers to hippocampus sample number Y from group X (group 1 is the control group which had daily i.p. injections of dH_2O; and 2, 3 and 4 treatment groups which had 0.1, 0.5 and 1 mg/kg B.W $CoCl_2$ daily i.p. injections for 28 days respectively). There are *n* = 4 samples per each control or treatment group, each group was later pooled into a single sample for RNA-Seq evaluation. Some of the samples could not be obtained and less samples than *n* = 4 were pooled.

Quality Check of RNA Samples from the Hippocampus					
**Samples**	**RNA Concentration (ng/µl)**	**Ratio [260/280]**	**Ratio [260/230]**	**Ratio [28S/18S]**	**RQI**
**Hippocampus Control (Hppc 1.1)**	184.4	2.1	2.18	1.41	9.6
**Hippocampus Control (Hppc 1.2)**	158.6	2.08	1.56	1.50	9.4
**Hippocampus Control (Hppc 1.3)**	372.6	2.08	2.28	1.13	9.3
**Hippocampus Control (Hppc 1.4)**	154.1	2.04	2.18	1.22	9.5
**Hippocampus 0.1 mg/kg BW (Hppc 2.1)**	109.2	2.05	2.15	1.24	9.3
**Hippocampus 0.1 mg/kg BW (Hppc 2.2)**	89.3	2.03	1.95	1.21	8.2
**Hippocampus 0.1 mg/kg BW (Hppc 2.3)**	-	-	-	-	-
**Hippocampus 0.1 mg/kg BW (Hppc 2.4)**	207.7	2.07	1.98	1.51	9.6
**Hippocampus 0.5 mg/kg BW (Hppc 3.1)**	-	-	-	-	-
**Hippocampus 0.5 mg/kg BW (Hppc 3.2)**	141.9	1.97	1.87	1.56	9.8
**Hippocampus 0.5 mg/kg BW (Hppc 3.3)**	174.2	2.07	2.27	1.61	9.7
**Hippocampus 0.5 mg/kg BW (Hppc 3.4)**	227.9	2.07	2.31	1.27	8.9
**Hippocampus 1 mg/kg BW (Hppc 4.1)**	-	-	-	-	-
**Hippocampus 1 mg/kg BW (Hppc 4.2)**	286.5	2.08	2.20	1.44	9.2
**Hippocampus 1mg/kg BW (Hppc 4.3)**	-	-	-	-	-
**Hippocampus 1 mg/kg BW (Hppc 4.4)**	-	-	-	-	-
**Pooled Samples**					
**Hippocampus - Control**	185.0	2.06	1.97	1.30	9.6
**Hippocampus - 0.1 mg/kg BW**	115.3	2.04	1.99	1.31	9.7
**Hippocampus - 0.5 mg/kg BW**	153.0	2.05	2.24	1.36	9.6
**Hippocampus - 1 mg/kg BW**	260.0	2.06	2.20	1.43	9.4

Table S4. MIQE checklist from the MIQE guidelines [1] for reproducibility and assessment of experimental RT-qPCR conditions. Essential information must be made available in the manuscript (E) while desirable information (D) is only made available if possible. Unless indicated otherwise, the protocol details required in the checklist have been presented in the Materials and Methods section of the paper.

Item to Check		Checklist
**Experimental Design**		
Definition of experimental and control groups	E	Yes
Number within each group	E	Yes
Assay carried out by the core or investigator's laboratory?	D	Yes
Acknowledgement of authors' contributions	D	-
**Sample**		
Description	E	Yes
Volume/mass of sample processed	D	Yes
Microdissection or macrodissection	E	Yes
Processing procedure	E	Yes
If frozen, how and how quickly?	E	Dissected samples were immediately submerged in RNALater and incubated at 4°C overnight. The day after, RNALater was removed and samples were frozen at -80°C
If fixed, with what, how quickly?	E	Not applicable
Sample storage conditions and duration especially for formalin-fixed, paraffin-embedded (FFPE) samples	E	Yes
**Nucleic Acid Extraction**		
Procedure and/or instrumentation	E	Yes
Name of kit and details of any modifications	E	Yes
Source of additional reagents used	D	Yes
Details of DNase or RNAse treatment	E	Not needed due to the presence of gDNA Eliminator Solution in the isolation kit (RNeasy Plus Universal Midi Kit (Qiagen, UK))
Contamination assessment (DNA or RNA)	E	No-reverse transcription (RT-) and no-template controls (NTC) were included
Nucleic acid quantification	E	Yes, through Nanodrop-2000c spectrophotometer
Instrument and method	E	Yes
Purity (A260/A280)	D	Yes (provided in Tables S1–3)
Yield	D	Yes (provided in Tables S1–3)
RNA integrity method/instrument	E	Yes, via Experion Automated Electrophoresis System
RNA integrity number/RNA quality indicator (RIN/RQI) or Cq of 3' and 5' transcripts	E	Yes (provided in Tables S1–3)
Electrophoresis traces	D	No
Inhibition testing (Cq dilutions, spike or other)	E	No, we used new reagents and the company's recommended concentrations
**Reverse Transcription**		
Complete reaction conditions	E	Yes
Amount of RNA and reaction volume	E	Yes
Priming oligonucleotide if using gene-specific priming (GSP) and concentration	E	Yes
Reverse transcriptase and concentration	E	Yes
Temperature and time	E	Yes
Manufacturer of reagents and catalogue numbers	D	Manufacturer is provided, but not the catalogue numbers.
Cqs with and without reverse transcription	D	Yes
Storage conditions of cDNA	D	Synthesised cDNA was kept at -20°C
**qPCR Target Information**		

Gene symbol	E	Yes
Sequence accession number	E	Yes
Location of amplicon	D	Yes
Amplicon length	E	Yes
In silico specificity screen (BLAST, and so on)	E	Yes
Pseudogenes, retropseudogenes or other homologs?	D	Primers retrieving pseudogenes from the target gene were used when there were no more options
Sequence alignment	D	No
Secondary structure analysis of amplicon	D	No
Location of each primer by exon or intron (if applicable)	E	No
What splice variants are targeted?	E	No
	**qPCR Oligonucleotides**	
Primer sequences	E	Yes
RTPrimerDB Identification Number	D	No
Probe sequences	D	No
Location and identity of any modifications	E	Not applicable
Manufacturer of oligonucleotides	D	Yes, Integrated DNA Technologies (IDT, Belgium)
Purification method	D	No
	**qPCR Protocol**	
Complete reaction conditions	E	Yes
Reaction volume and amount of cDNA/DNA	E	Yes
Primer, (probe), Mg++ and deoxynucleoside triphosphate (dNTP) concentrations	E	Yes
Polymerase identity and concentration	E	Yes
Buffer/kit identity and manufacturer	E	Yes
Exact chemical constitution of the buffer	D	No (not provided by manufacturer)
Additives (SYBR Green I, DMSO, and so forth)	E	No
Manufacturer of plates/tubes and catalog number	D	Yes
Complete thermocycling parameters	E	Yes
Reaction setup (manual/robotic)	D	Manual setup
Manufacturer of qPCR instrument	E	Yes
	**qPCR Validation**	
Evidence of optimisation (from gradients)	D	No
Specificity (gel, sequence, melt, or digest)	E	Yes, melt curve
For SYBR Green I, Cq of the NTC	E	Yes
Calibration/Standard curves with slope and y intercept	E	No
PCR efficiency calculated from slope	E	PCR efficiencies were calculated but not applied due to the limited range of the standard curve for some primers meaning that the Cts from the most diluted samples of the 5-fold serial dilution (Ct>26) were close to the reliable limit of quantification (Ct~40) in the case of low copy number genes.
Confidence interval (CI) for PCR efficiency or standard error (SE)	D	No
r2 of standard curve	E	No
Linear dynamic range	E	No
Cq variation at lower limit	E	No
Confidence intervals throughout range	D	No

Evidence for limit of detection (LOD)	E	No
If multiplex, efficiency and LOD of each assay.	E	Not applicable
**Data Analysis**		
qPCR analysis program (source, version)	E	Yes
Cq method determination	E	Yes
Outlier identification and disposition	E	No data points were excluded as outliers
Results of NTCs	E	Cts of non-template controls were labelled as 'Undetermined' by the software meaning that they were below the detection limit.
Justification of number and choice of reference genes	E	Yes
Description of normalisation method	E	Yes
Number and concordance of biological replicates	D	Yes
Number and stage (RT or qPCR) of technical replicates	E	Yes
Repeatability (intra-assay variation)	E	Yes
Reproducibility (inter-assay variation, %CV)	D	No
Power analysis	D	Not applicable
Statistical methods for result significance	E	Yes
Software (source, version)	E	Yes
Cq or raw data submission using RDML	D	No

Table S5: Criteria for the design of the primers selected through NCBI Primer-BLAST tool. Bp refers to base pairs.

Primer Design Condition	Design Criteria
PCR primer size (bp)	130–150
Primer melting temperature (Tm)	59–61 °C
Max. melting temperature difference between forward and reverse primer	≤1 °C
Stability at 3' primer end	Max. 3 Gs or Cs bases in the last 5bp
Long runs of repeated bases	Max. 4 contiguous repeated bases
Conditions for genomic DNA exclusion	Exon junction span (forward primer or reverse primer) or intron inclusion
GC content (%)	40–60%
Specificity	Specific to targets, only predicted transcript variants of targets allowed when no option.

Table S6: Primer sequences of targeted genes designed for the *in vivo* dose-response experiment. Gene symbol, accession number, amplicon length and calculated primer melting temperature (Tm) are supplied as obtained from NCBI Primer-BLAST.

Gene	Accession no.		Primer Sequence (5'-3')	Length (bp)	Tm
Ttr	NM_012681.2	F	GGCTCACCACAGATGAGAAGT	149	59.72
		R	GGTGTAGTGGCGATGACCAG		60.46
Tnf	NM_012675.3	F	ACGTCGTAGCAAACCACCAA	132	60.18
		R	AGATAAGGTACAGCCCATCTGC		59.63
Akap14	NM_021703.1	F	TCAGTTTGTGGAAGAAGCCAGA	141	59.83
		R	GCATAGTACACCCAGCGGTT		60.11
Spata18	NM_199374.2	F	CCCAGGTTCAAGACGATCTGAC	139	60.68
		R	TCCTCCTGGGCTTGAAGAGAT		60.27
Kl	NM_031336.1	F	TCCCTGTGACTTTGCTTGGG	141	60.18
		R	TTGGCTACAACCCCGTCTAC		59.39

Table S7: Fast PCR thermal cycling steps based on PowerUpTM SYBRTM Green Master Mix instructions for StepOnePlus Real-Time PCR system.

RT-qPCR Stage	Step	T (°C)	Duration	Cycles
Holding stage	UDG activation	50	2 minutes	Hold
	Dual-Lock DNA polymerase	95	2 minutes	Hold
Cycling stage	Denature	95	3 seconds	40
	Anneal/extend (data collection)	60	30 seconds	
Melting curve	Denature	95	15 seconds	1
	Anneal/extend	60	1 minute	
	Denature (data collection)	95	15 seconds	

Table S8: Primer sequences of control genes, with gene symbol, accession number, amplicon length and calculated primer melting temperature (Tm) as supplied by NCBI Primer-BLAST.

Gene	Accession no.		Primer Sequence (5'-3')	Length (bp)	Tm
Ywhaz	NM_013011.3	F	GAGTCGTACAAAGACAGCACG	131	59.29
		R	AAAGGTTGGAAGGCCGGTTA		59.52
Tbp	NM_001004198.1	F	ACTTCGTGCCAGAAATGCTGA	140	60.54
		R	TGGATTGTTCTTCACTCTTGGCT		60.18
Pes1	NM_001044228.1	F	GTACAAGGTGTTTGTCCGGAAG	148	59.45
		R	GTCACGCAACGCATCGATAA		59.36

Selection of Reference Genes for the Normalisation of Target Gene Expression through RT-qPCR

The candidate reference genes for normalisation of gene expression data were *Ywhaz*, *Pes*, and *Tbp*. Figure S1 shows the RefFinder ranking results for the pref. cortex tissue from the dose-response experiment while Figure S2 shows the results for the hippocampus. *Pes1* gene was selected for the pref. cortex and *Ywhaz* for the hippocampus.

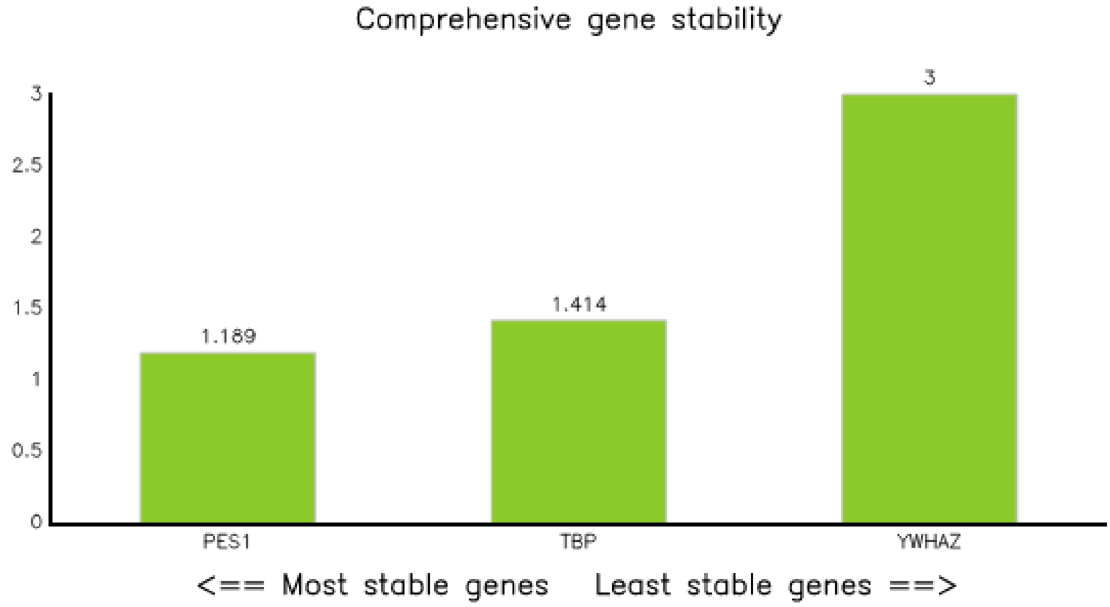

Figure S1. RefFinder ranking of three reference genes Ct values in pref. cortex samples from the *in vivo* dose-response experiment. Only the samples from the rats dosed with i.p. injections for 28 days with dH20 (controls) and 1mg/kg B.W. $CoCl_2$ (treatment group) were analysed. The candidate reference genes were *Ywhaz*, *Pes1* and *Tbp*. The most stable and gene selected for further RT-qPCR assays in the pref. cortex samples of the time-response experiment is *Pes1*.

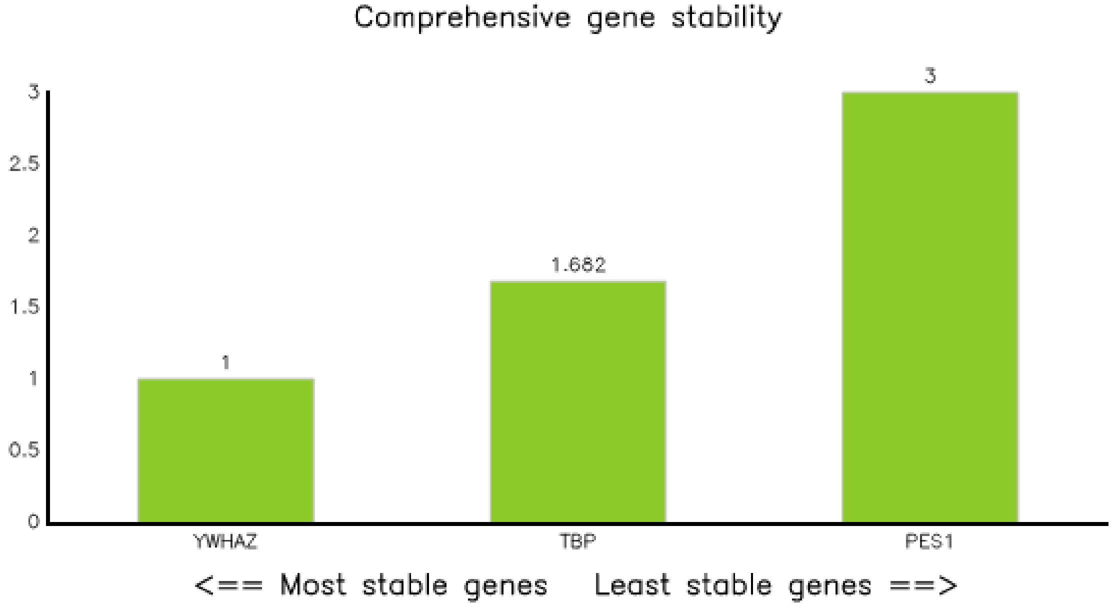

Figure S2: RefFinder ranking of three reference genes Ct values in hippocampus samples from the *in vivo* dose-response experiment. Only the samples from the rats dosed with i.p. injections for 28 days with dH_2O (controls) and 1mg/kg B.W. $CoCl_2$ (treatment group) were analysed. The candidate reference genes were *Ywhaz*, *Pes1* and *Tbp*. The most stable and gene selected for further RT-qPCR assays in the hippocampus samples of the time-response experiment is *Ywhaz*.

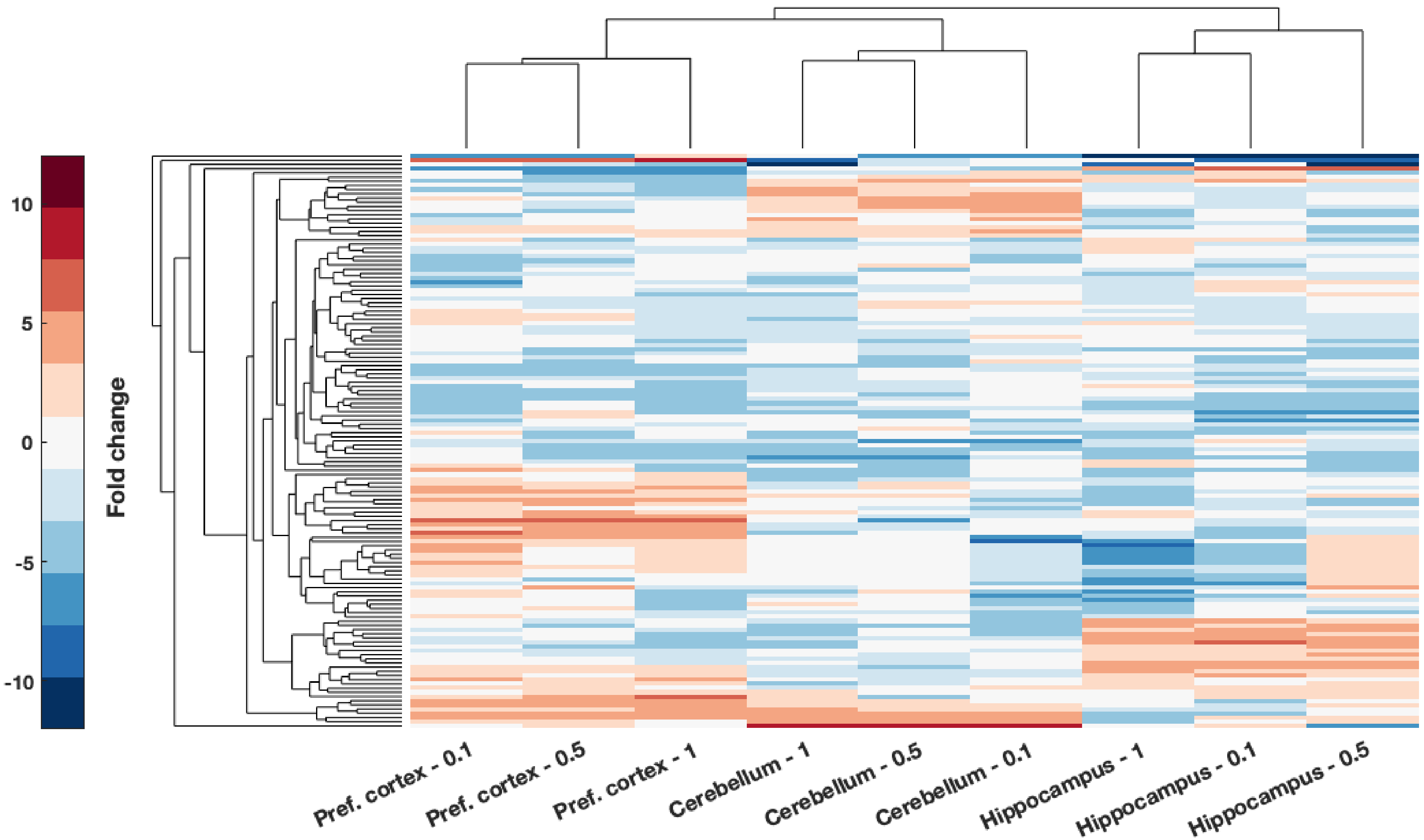

Figure S3: Hierarchical clustering of DEGs over 2-fold-change from RNA-Seq data obtained from the comparison of pref. cortex, cerebellum and hippocampus of rats treated with three concentrations of cobalt against those of controls treated with dH_2O. RNA was isolated from brain tissues of S.D. male rats treated with daily i.p. injections for 28 days with different concentrations of cobalt: 0.1, 0.5 and 1 mg/kg B.W. $CoCl_2$. Samples analysed in each group were pooled ($n' = 1$) from $n = 4$, except in the case of the hippocampus of rats treated with 0.5 mg/kg B.W. $CoCl_2$, $n = 3$, and 1 mg/kg B.W. $CoCl_2$, $n = 1$. Dendrogram heights display the Euclidian distance between DEGs pairs, and colour bar shows the fold change.

Gene expression Validated by RT-qPCR

Transthyretin (*Ttr*) and *Akap14* were chosen as markers of choroid plexus contamination. *Tnf* and *Spata18* are markers of inflammation and mitochondrial damage, respectively. The highest dose of cobalt treatment (1 mg/kg B.W. $CoCl_2$) was selected to evaluate gene expression against the control group in these targets.

Figure S4 shows the fold change according to RT-qPCR and RNA-Seq, which in general are approximatively similar. However, that is not the case for *Tnf* and *Ttr*. The expression of none of the genes selected was significantly different in the treatment groups from that of the controls. The ΔC_T values shown in Figure S5 display dissimilar averages in the case of *Spata18*, *Akap14* and *Kl*. However, they are not sufficient to declare statistical significance. For the hippocampus, the fold change of *Kl* for cobalt treatment groups 0.5 and 1 mg/kg B.W. is shown, although only the 0.5 mg/kg B.W. group is displayed as ΔC_T values. That is because there was only one sample available of the hippocampus for the 1 mg/kg B.W. treatment group due to limited tissue availability after dissection.

The fold-change values observed from the RT-qPCR results (Figure S4) do not always correspond with those obtained from the RNA-Seq data, especially in the case of *Tnf* and *Ttr* in the pref. cortex. It is known that a few genes will produce inconsistent results across RT-qPCR and RNA-Seq platforms [2]. Figure S5 shows that the ΔC_T values of individual samples are within a broad range and the standard errors of the mean (SEMs) are wide. As a result, none of the genes selected for RT-qPCR screening had significant changes in transcript levels. However, the ΔC_T mean and SEM also indicate a biological change in the amount of target mRNA transcripts in the case of *Spata18* and *Akap14*. This lack of significance could have its roots in the technical differences between RT-qPCR and RNA-Seq, the low number of replicates used ($n = 4$), and the consequences of using pooled samples in this research design setting [3]. Despite the lack of significance, the fold expression of evaluated DEGs from RNA-Seq and RT-qPCR in this study do correlate in general, as in other studies comparing the two technologies [2].

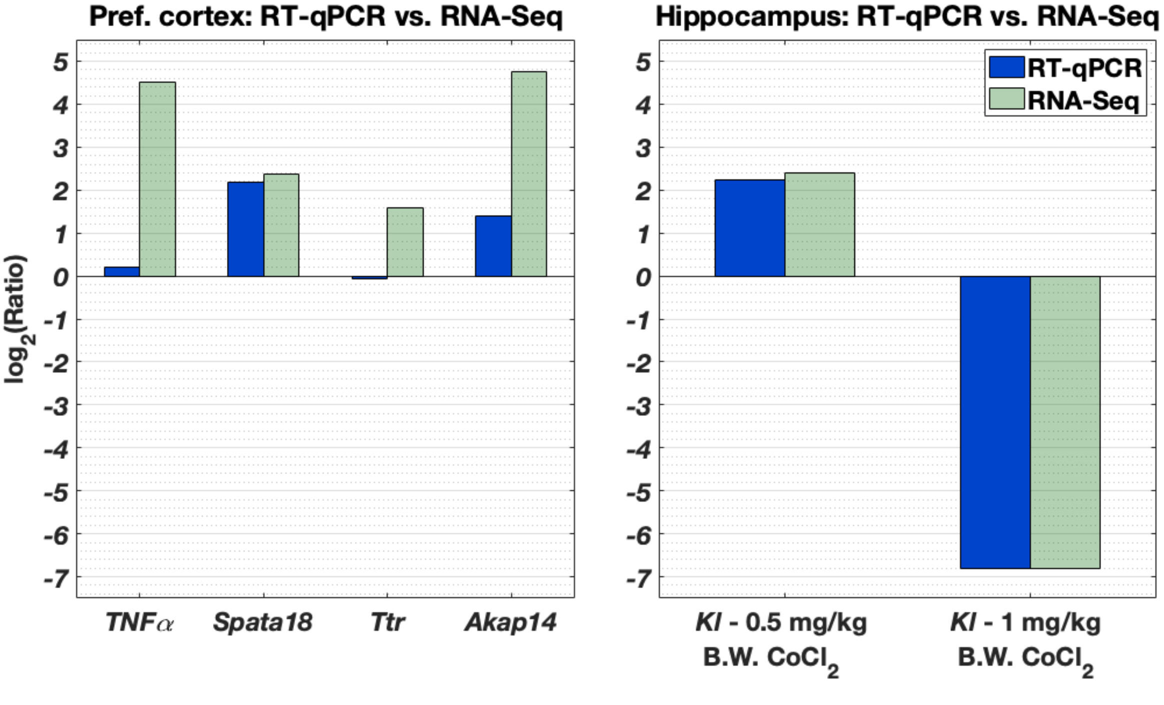

Figure S4: Fold change mRNA gene expression levels obtained from RNA-Seq (green) and RT-qPCR (blue) of *Tnf, Spata18, Ttr, Akap14* (pref. cortex; $n' = 1$ of $n = 4$ pooled samples) and *Kl* (Klotho; hippocampus; $n' = 1$ from $n = 4$ control group, $n = 3$ in 0.5 mg/kg B.W. $CoCl_2$ treatment group, or $n = 1$ in treatment 1 mg/kg B.W. $CoCl_2$ pooled samples). RT-qPCR normalisation was against *Pes1* in the pref. cortex and *Ywhaz* in the hippocampus. The y-axes show the logarithm of the fold change (aka ratio).

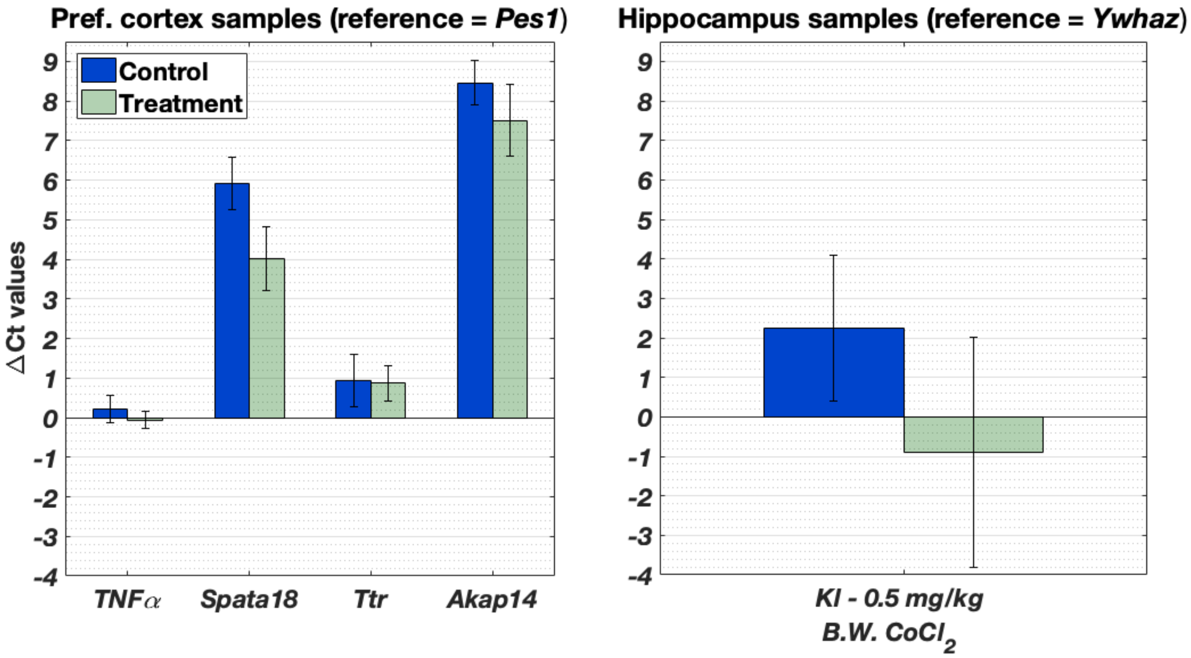

Figure S5: Quantification of *Tnf, Spata18, Ttr, and Akap14* ΔCT values (CT target gene – CT internal control) in the pref. cortex and *Kl* in the hippocampus through RT-qPCR. The internal control for pref. cortex is *Pes1*, and *Ywhaz* for the hippocampus. Data are displayed as mean ± SEM (n = 4 for pref. cortex and control group in hippocampus, n = 3 for treatment group in hippocampus). No significant changes were found between 0.5 (hippocampus) or 1 (pref. cortex) mg/kg B.W. $CoCl_2$ treatment groups and control groups after 28-days i.p. injection treatment by independent-samples t-test ($p < 0.05$).

References

1. Bustin, S.A.; Benes, V.; Garson, J.A.; Hellemans, J.; Huggett, J.; Kubista, M.; Mueller, R.; Nolan, T.; Pfaffl, M.W.; Shipley, G.L. The MIQE Guidelines: Minimum Information for Publication of Quantitative Real-Time PCR Experiments. *Clin. Chem.* **2009**, *55*, 611–622, https://doi.org/10.1373/clinchem.2008.112797.
2. Everaert, C.; Luypaert, M.; Maag, J.L.; Cheng, Q.X.; Dinger, M.E.; Hellemans, J.; Mestdagh, P. Benchmarking of RNA-sequencing analysis workflows using whole-transcriptome RT-qPCR expression data. *Sci. Rep.* **2017**, *7*, 1559, https://doi.org/10.1038/s41598-017-01617-3.
3. Assefa, A.T.; Vandesompele, J.; Thas, O. On the utility of RNA sample pooling to optimize cost and statistical power in RNA sequencing experiments. *BMC Genom.* **2020**, *21*, 312, https://doi.org/10.1186/s12864-020-6721-y.
